# Social determinants of where people die: A study of moderators and mediators using linked UK Census and mortality data

**DOI:** 10.1016/j.ssmph.2025.101784

**Published:** 2025-03-21

**Authors:** J.M. Davies, K.C. Chua, M. Maddocks, F.E.M. Murtagh, K.E. Sleeman

**Affiliations:** a Cicely Saunders Institute, Department of Palliative Care, Policy and Rehabilitation, Faculty of Nursing, Midwifery and Palliative Care, King's College London, UK; b Centre for Implementation Science, Health Service & Population Research, Institute of Psychiatry, Psychology and Neuroscience, King's College London, UK; c Wolfson Palliative Care Research Centre, Hull York Medical School, University of Hull, Hull, UK

## Abstract

**Background:**

Where people die depends on many factors and is important to the quality of end-of-life care. Many people prefer to avoid end-of-life hospital admissions and yet hospital remains the most common place of death across high-income countries and is more likely for people who live in more deprived areas. This study examines moderators and mediators of socioeconomic inequality in place of death.

**Methods:**

We used census data linked to mortality data for people who died in England and Wales between 2011 and 2017 to investigate the association between area-based income deprivation and death in hospital versus home, hospice, and care home. We tested moderators including age, sex, ethnicity, underlying cause of death and region, and mediating pathways through housing deprivation, living alone, and worse health.

**Results:**

Among 34,230 decedents, after adjusting for age and sex, the proportion of deaths in hospital was higher in more deprived areas; 52.4 % (95 % CI: 51.2 %–53.6 %) and 46.7 % (45.5 %–48.0 %) for people living in the most and least deprived areas, respectively. This association was moderated by underlying cause of death; a social gradient was observed for deaths from cancer, dementia and ‘other’ causes but not for people who died from respiratory, cardiovascular, and sudden causes (F = 43.81; df (20), p = 0.0016). In a subsample of people who died from cancer, people living in the most deprived areas were more likely to live alone (36 % (95 % CI 30 %–41 %)) than those in the least deprived areas (19 % (15 %–23 %)), and this partly explained why they were more likely to die in hospital, accounting for 12.2 % of the total effect of income deprivation on death in hospital.

**Conclusion:**

This study contributes novel findings that deepen our understanding of socioeconomic inequality in place of death. Improving support for people living alone in more deprived areas is identified as a potential way to reduce inequalities in place of death.

## Highlights

• Where people die is important to patients and families, most prefer to avoid hospitalisation, but hospital is the most common place of death.
• People who live in more deprived areas are more likely to die in hospital, but this study shows that this depends on cause of death.
• For people who died from cancer, those living in more deprived areas were more likely to live alone and this partly explains why they were more likely to die in hospital.

## 1 Background

Where people die and preferences for place of care and place of death depend on many social and clinical factors (Gomes et al., 2013). Many people prefer to avoid hospitalisations towards the end of life (Gomes et al., 2012). Yet, despite a declining trend in the proportion of hospital deaths, hospital remains the most common place of death in most high income countries (Lopes et al., 2024). A recent umbrella review found that home is the most preferred place of end-of life care and death for patients and family members, but with substantial minorities preferring hospice, palliative care facilities, and hospital (Pinto et al., 2024). Reasons for wanting to die at home include the possibility of being surrounded by friends and family and having more autonomy and dignity (Pinto et al., 2024). Reasons against wanting to die at home include a lack of support or inability to provide care at home (Pinto et al., 2024; Dittborn et al., 2021). Some people may prefer to die in hospital seeing it as a safe place with better symptom management and a way to relieve family burden (Pinto et al., 2024; Caroline et al., 2016).

Socioeconomic inequality in place of death is well described (Davies et al., 2018, 2019). In high-income countries, people living in more deprived areas are consistently found to be more likely to die in hospital (compared to home or hospice) (Davies et al., 2019), and other factors associated with death in hospital include non-white ethnicity and non-cancer cause of death (Gao, Verne, et al., 2014). In the UK, the association between living in a more deprived area and the higher risk of death in hospital (compared to home) was first described more than 20 years ago for people who died from cancer (Higginson et al., 1999) and subsequently in other patient populations (Gao, Ho, et al., 2014). Yet, to our knowledge, no studies have investigated whether this inequality is uniform across groups in society. We know very little about the intersection between socioeconomic position and other factors such as age, sex, ethnicity, and cause of death, and there has been little empirical work on the causes of inequality in place of death.

To address inequality in place of death we need a better understanding of who experiences inequality and what the causes of inequality are so that we can target policies and interventions more effectively and ensure that new services and interventions do not exacerbate existing inequalities. One previous study has tested potential explanatory pathways between wealth and use of hospital care towards the end of life (Davies et al., 2021). It found that worse health partly explained why people with lower wealth had more hospital admissions but none of the pathways tested explained the effect of lower wealth on death in hospital (Davies et al., 2021). Poor quality housing (Hansford et al., 2022a) and a lack of social support (Grundy et al., 2004; Hansford et al., 2022b) are other possible mechanisms that could explain social inequality in place of death.

The first aim of this study is to understand *for whom* is low socioeconomic position a predictor of death in hospital (versus home, hospice or care home), testing age, sex, ethnicity, underlying cause of death and region of residence as potential moderators. The second aim is to understand for groups where there is an association, *why* is low socioeconomic position associated with place of death, testing mediating pathways through housing deprivation, living alone, and worse health.

## 2 Methods

### 2.1 Study design, data, and participants

This is a retrospective observational cohort study in two parts, using data from the Office for National Statistics Longitudinal Study for England and Wales (ONS LS), which contains linked census and mortality data for a 1% sample of the population of England and Wales. First, we tested moderators of the relationship between area-based deprivation and death in hospital using a sample of ONS LS members who completed the Census in 2011 and subsequently died between 2011 and 2017. Second, we tested mediators of the relationship between area-based deprivation and death in hospital using a sample of ONS LS members who completed the Census in 2011 and subsequently died within 12 months of completing the Census.

### 2.2 Study variables

The outcome was death in hospital (compared to home, hospice, or care home for part one (testing moderators) and compared to home only for part two (testing mediators)). People who died in ‘other’ locations or ‘elsewhere’ or who had missing place of death were excluded from the analysis. We examined one measure of socioeconomic position, five potential moderators and three potential mediators (Table 1 has a summary of the variables we used).

**Table 1:** Summary of variables.

**Socioeconomic position exposure**	**Area-based income deprivation:** Our main exposure variable, used as a proxy for individual level socioeconomic position, was area-based income deprivation, measured using the income domain of the Index of Multiple Deprivation (IMD) for England and Wales 2015 (Tom Smith et al., 2015). The overall IMD, contains seven domains of deprivation including: income, employment, health, education, crime, barriers to housing, and environment. To avoid overlap with our housing deprivation mediator, we used the income domain rather than the overall IMD. The income domain of the IMD measures the proportion of people in each neighbourhood (Lower Super Output Area (LSOA)) in receipt of certain means-tested benefits, including: child, employment, and pension benefits. We used national quintiles of income deprivation linked to the decedent's LSOA of residence at the 2011 Census (1 is most deprived).
**Moderators (part 1)**	*A moderator is a variable that changes the relationship between the exposure and the outcome.* **Age at death:** In completed years, from the mortality record. **Sex:** Response to 2011 census question: ‘What is your sex?’, options are: male, or female. **Ethnicity:** Self-reported on the 2011 Census and grouped into 6 categories: White British, White other, Mixed ethnicity, South Asian (including Indian, Pakistani, or Bangladeshi), Black (including black African and black Caribbean), ‘other’ ethnicity. **Underlying cause of death:** From the mortality record, grouped into 6 categories: cancer, dementia, cardiovascular, respiratory, other causes, and sudden causes. Sudden causes were defined following an established method of classifying deaths that are most likely to have been sudden and unexpected (Murtagh et al., 2013). **Region of residence:** Linked to the decedent's place of residence at the 2011 Census. Grouped into 9 regions of England, plus Wales.
**Mediators (part 2)**	*A mediator is a variable that sits on the causal pathway from the exposure to the outcome, the exposure causes the mediator which in turn causes the outcome.* **Living alone:** A binary variable indicating whether the deceased was living alone at the time of completing the 2011 Census. **Housing deprivation:** We derived a measure of housing deprivation from the following 2011 Census indicators: • Overcrowded household: defined as having fewer rooms than ‘required’. A one-person household is assumed to require three rooms: two common rooms and one bedroom. Households with two residents or more are assumed to require two common rooms plus one bedroom for each of the following: each couple, each single adult, two adolescents (aged 10 to 20) of the same sex, two children (aged 9 year or under) regardless of sex, and any remaining child. • Household with no central heating. • Household that is not self-contained: defined as some or all the household rooms being shared with one or more other households. • Not living in a house or bungalow: i.e. living in a flat, tenement block, bed-sit, commercial building, caravan or other mobile or temporary structure. Participants were assigned a score from 0 to 4 based on how many of the above indicators were present. **General health:** In response to the 2011 Census question: ‘How is your health in general?’ Reported on a 5-point scale: Very good (1), good (2), fair (3), bad (4), very bad (5).

## 3 Analysis

The stages of analysis are described below and summarised in a Supplementary Table 1.

### 3.1 Part 1: Testing potential moderators

#### 3.1.1 Descriptive and preliminary analysis

We described the variables for the overall sample and by quintiles of area-based income deprivation using frequencies, proportions, and medians. To understand the relationship between the outcome (place of death) and our main exposure (area-based income deprivation), we used a multinomial logit regression to predict 4 un-ordered categories of place of death (home, hospice, care home; with hospital as the reference category), adjusted for age, sex, and underlying cause of death.

Cause of death is an important determinant of place of death, for example people dying from cancer are more likely to die at home or in hospice than people dying from respiratory disease (Gao, Ho, et al., 2014). Cause of death is also associated with socioeconomic position, for example people living in more deprived areas are less likely to die from cancer and more likely to die from respiratory disease (NEoLCIN, 2012). Therefore, to understand this important association in more detail for our sample, we have reported the predicted proportions for each underlying cause of death, by level of area-based deprivation from a multinomial logit regression, adjusted by age and sex.

#### 3.1.2 Moderator analysis

We ran separate models for each potential moderator (adjusted for age and sex) and added an interaction term between area-based income deprivation and the moderator. Then we compared the model with the interaction term to the nested model with no interaction using the Wald test *F* statistic. To help with interpretation, we derived marginal effects and graphically depicted the interactions using line graphs. We used Poisson regression models to examine the risk of death in hospital versus home, hospice, or care home. We chose Poisson models because risk ratios are arguably more easily interpreted than odds ratios. We applied robust standard errors to account for heteroskedasticity.

### 3.2 Part 2: Multiple mediator model

**Fig. 1:**
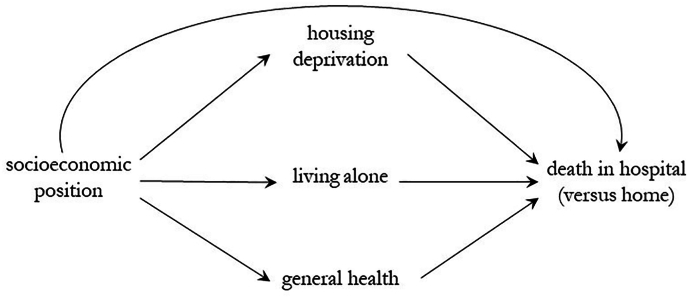
Direct Acyclic Diagram (DAG) depicting the hypothesised pathways∗ between socioeconomic position and death in hospital (versus home) ∗Each line with an arrow represents a relationship that can be estimated with a regression. Age and sex are covariates with arrows to each of the mediators and the outcome (not shown here).

Our multiple mediator model is visually depicted in Fig. 1. We tested three potential pathways that may explain why people with lower socioeconomic position are more likely to die in hospital i) through worse health, because people who live in more deprived areas have worse health and therefore may have a higher need for hospital care towards the end of life (Davies et al., 2021); ii) through living alone, because people living in more deprived areas may be more likely to live alone, which may be associated with a higher risk of death in hospital (Grundy et al., 2004), and iii) through housing, because people living in more deprived areas are more likely to live with poor housing quality, a known social determinant of health, which may also be important to place of death (Richards, et al., 2024; Hansford et al., 2022a).

Informed by the moderation analysis, we restricted the model to a subsample where we had established a relationship between income deprivation and death in hospital. We applied several other restrictions to the sample: i) we restricted the sample to people who died within 12 months of completing the 2011 Census so that the mediators were more likely to reflect living conditions at death; ii) we excluded people living in communal establishments (i.e. care or nursing homes) at the time of the 2011 Census and people who died in a care home because two of the mediators (housing deprivation and living alone) are not applicable to people living in communal establishments; iii) we excluded people who died in a hospice because our theoretical model is focused on hospital versus home as an outcome.

In preliminary analysis, to understand the relationships between variables we regressed the outcome and each mediator on the primary exposure variable, area-based income deprivation, adjusting for age and sex.

For the multiple mediation model, we used the Karlson, Holm and Breen (KHB) mediation method, which extends standard mediation techniques developed with linear regression models to nonlinear outcomes (Breen et al., 2013). A mediation analysis compares coefficients across models with and without mediators and, decomposes i) the direct effect of the exposure on the outcome from ii) the indirect effect of the exposure on the outcome via the mediators. In non-linear models, this comparison of coefficients across different models is complicated by variation in the scale parameters of the different exposures. The KHB method addresses the ‘scaler problem’ by separating out true changes in coefficients from changes in coefficients due to variation in scaling. This method has been shown to produce valid estimates across a range of non-linear models (Kohler et al., 2011; Williams & Jorgensen, 2023).

We report coefficients from the *Reduced Model* (i.e. the model reporting the total effect of the exposure) and the *Full Model* (i.e. the model reporting the direct effects of the exposure and the mediators, and the indirect effects of the exposure via the mediators). Our analysis used logit models (the KHB method has not yet been validated for Poisson models) and we have reported coefficients, 95 % confidence intervals and the proportion of the confounding explained by each of the mediators.

All analysis was carried out in Stata v.17(StataCorp 2021), code is available from: https://github.com/joannamariedavies/ONS_LS.

#### 3.2.1 Sensitivity analysis

To retain statistical power, in our main analysis we treated quintiles of area-based income deprivation as a continuous variable. In a sensitivity analysis we treated this main exposure as a categorical variable and discuss differences in the results.

#### 3.2.2 Patient and public involvement

This study has involved patients and the public throughout (see our report summarising PPI workshops used to help with the interpretation of data) (Brittain et al., 2024).

## 4 Results

**Fig. 2:**
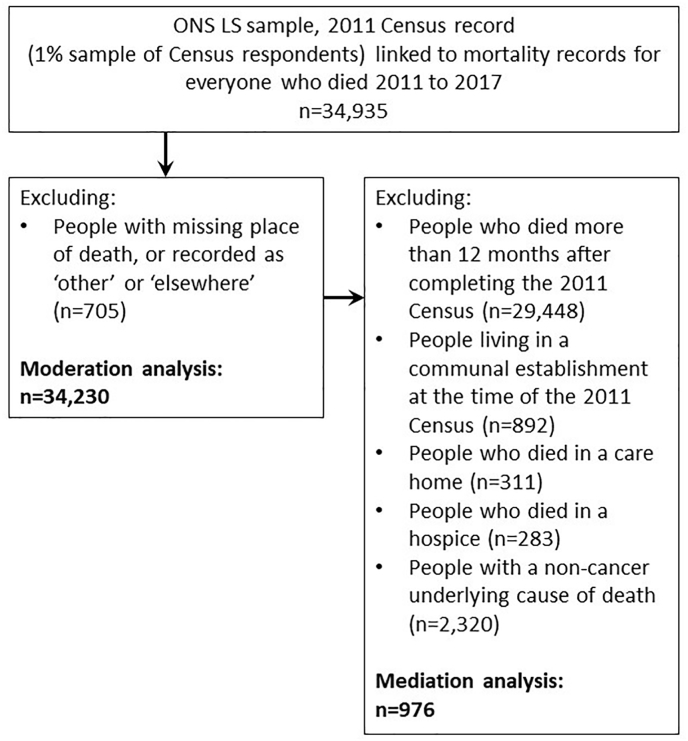
Flow chart of participants included in and excluded from the study (data source: Office for National Statistics Longitudinal Study (ONS LS)).

Fig. 2 describes the participants included in the moderation and mediation analyses.

### 4.1 Part 1: Moderator analysis

We included 34,230 people in the moderator analysis, Table 2 describes the characteristics of the sample. Compared to people living in less income deprived areas, people living in more deprived areas died at younger ages, a larger proportion died from respiratory disease, a larger proportion were from ethnic minority groups, and a larger proportion lived in the northern regions and West Midlands of England.

**Table 2:** Characteristics of participants included in the moderation analysis, overall and by level of area-based income deprivation.

	overall	1 (most income deprived)	2	3	4	5 (least income deprived)
N	34230	6903	7022	7219	7004	6077
**age at death, median (IQR**^a^**)**	82 (72, 89)	79 (69, 87)	82 (72, 89)	82 (73, 89)	83 (74, 89)	83 (74, 89)
**gender**						
Male	16338 (47.7 %)	3290 (47.7 %)	3334 (47.5 %)	3352 (46.4 %)	3336 (47.6 %)	3025 (49.8 %)
Female	17892 (52.3 %)	3613 (52.3 %)	3688 (52.5 %)	3867 (53.6 %)	3668 (52.4 %)	3052 (50.2 %)
**ethnicity**						
white British	31281 (91.4 %)	5980 (86.6 %)	6256 (89.1 %)	6681 (92.5 %)	6618 (94.5 %)	5741 (94.5 %)
white other	1233 (3.6 %)	285 (4.1 %)	292 (4.2 %)	243 (3.4 %)	210 (3.0 %)	203 (3.3 %)
mixed ethnicity	138 (0.4 %)	38 (0.6 %)	43 (0.6 %)	31 (0.4 %)	13 (0.2 %)	13 (0.2 %)
Indian, Pakistani, Bangladeshi	902 (2.6 %)	320 (4.6 %)	252 (3.6 %)	161 (2.2 %)	103 (1.5 %)	66 (1.1 %)
Black	394 (1.2 %)	207 (3.0 %)	96 (1.4 %)	53 (0.7 %)	25 (0.4 %)	13 (0.2 %)
other ethnicity	267 (0.8 %)	71 (1.0 %)	79 (1.1 %)	45 (0.6 %)	34 (0.5 %)	38 (0.6 %)
missing	15 (<1 %)	2 (<1 %)	4 (0.1 %)	5 (0.1 %)	1 (<1 %)	3 (<1 %)
**underlying cause of death**						
cancer	9880 (28.9 %)	1946 (28.2 %)	1904 (27.1 %)	2061 (28.5 %)	2106 (30.1 %)	1860 (30.6 %)
dementia	3691 (10.8 %)	655 (9.5 %)	768 (10.9 %)	786 (10.9 %)	795 (11.4 %)	686 (11.3 %)
cardiovascular	8552 (25.0 %)	1664 (24.1 %)	1790 (25.5 %)	1845 (25.6 %)	1726 (24.6 %)	1526 (25.1 %)
respiratory	2028 (5.9 %)	581 (8.4 %)	459 (6.5 %)	415 (5.7 %)	329 (4.7 %)	244 (4.0 %)
other	1759 (5.1 %)	353 (5.1 %)	341 (4.9 %)	380 (5.3 %)	358 (5.1 %)	327 (5.4 %)
sudden causes	8320 (24.3 %)	1704 (24.7 %)	1760 (25.1 %)	1732 (24.0 %)	1690 (24.1 %)	1434 (23.6 %)
**region of residence**						
North East	1784 (5.2 %)	693 (10.0 %)	393 (5.6 %)	258 (3.6 %)	238 (3.4 %)	202 (3.3 %)
North West	4612 (13.5 %)	1432 (20.7 %)	885 (12.6 %)	748 (10.4 %)	824 (11.8 %)	722 (11.9 %)
Yorkshire and The Humber	3475 (10.2 %)	927 (13.4 %)	688 (9.8 %)	660 (9.1 %)	658 (9.4 %)	542 (8.9 %)
East Midlands	2841 (8.3 %)	510 (7.4 %)	585 (8.3 %)	680 (9.4 %)	629 (9.0 %)	437 (7.2 %)
West Midlands	3445 (10.1 %)	988 (14.3 %)	633 (9.0 %)	656 (9.1 %)	698 (10.0 %)	469 (7.7 %)
East of England	3678 (10.7 %)	379 (5.5 %)	763 (10.9 %)	967 (13.4 %)	855 (12.2 %)	714 (11.7 %)
London	3428 (10.0 %)	860 (12.5 %)	984 (14.0 %)	682 (9.4 %)	527 (7.5 %)	375 (6.2 %)
South East	5238 (15.3 %)	375 (5.4 %)	905 (12.9 %)	1146 (15.9 %)	1233 (17.6 %)	1579 (26.0 %)
South West	3609 (10.5 %)	370 (5.4 %)	729 (10.4 %)	937 (13.0 %)	936 (13.4 %)	637 (10.5 %)
Wales	2120 (6.2 %)	369 (5.3 %)	457 (6.5 %)	485 (6.7 %)	406 (5.8 %)	400 (6.6 %)
**place of death**						
home	8005 (23.4 %)	1680 (24.3 %)	1552 (22.1 %)	1673 (23.2 %)	1633 (23.3 %)	1466 (24.1 %)
hospital	16929 (49.5 %)	3642 (52.8 %)	3689 (52.5 %)	3461 (47.9 %)	3297 (47.1 %)	2839 (46.7 %)
hospice	1951 (5.7 %)	356 (5.2 %)	337 (4.8 %)	400 (5.5 %)	455 (6.5 %)	402 (6.6 %)
care home	7345 (21.5 %)	1225 (17.7 %)	1444 (20.6 %)	1685 (23.3 %)	1619 (23.1 %)	1370 (22.5 %)

a Interquartile Range (IQR).

The multinomial logistic regression (Supplementary Table 2) shows that compared to hospital, people who lived in areas with more income deprivation were less likely to die at home, in hospice, and in a care home, than people living in less income deprived areas. This result supports the grouping of hospital versus all other locations that is used in the remainder of the moderation analysis. Supplementary Table 3, shows that after adjusting for age and sex, compared to people living in the least income deprived areas, fewer people living in the most deprived areas died from cancer and more died from respiratory disease.

**Fig. 3:**
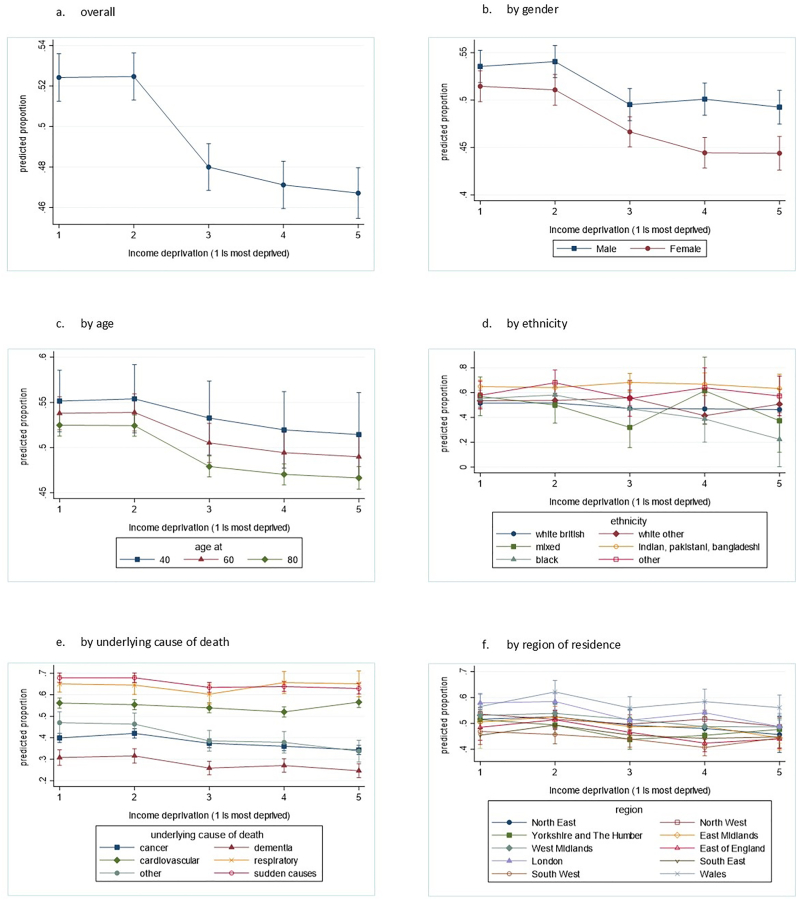
Predicted proportion (from Poisson models adjusted for age and sex) of deaths in hospital (versus home, hospice, and care home) by level of area-based income deprivation, and marginal effects by the different moderators: age, gender, ethnicity, underlying cause of death, and region of residence (data source: Office for National Statistics Longitudinal Study (ONS LS)) (note the variation in y axis scale across the graphs).

In the overall sample, the association between level of area-based income deprivation and death in hospital (versus home, hospice, and care home) is negative but non-linear (Fig. 3a), indicating that a categorical measure of deprivation is most appropriate for this data.

Supplementary tables 4.1 to 4.5 report the interaction effects, and Supplementary Table 5 shows the model fit statistics comparing the models with an interaction term between area-based income deprivation and each of the moderators tested, to the nested model with no interaction.

Of the potential moderators tested only underlying cause of death had a statistically significant interaction with area-based deprivation. Fig. 3e shows that for people who died from cancer, dementia, and ‘other’ causes, those who were living in more income deprived areas were more likely to die in hospital. This association between area-based deprivation and death in hospital is less clear for people who died from sudden causes and is not observed for people who died from cardiovascular and respiratory disease.

### 4.2 Part 2: Mediation modelling

The moderation analysis established a relationship between income deprivation and death in hospital for people who died from cancer, dementia and from other causes. On this basis we restricted the mediation analysis to people who died from cancer to focus the model on a relatively homogenous group (see Fig. 2 for a list of other exclusions). We included 976 participants in the mediation model, Table 3 reports the sample characteristics. For the housing deprivation variable, 770 people (78.9 %) had no indicators of housing deprivation, 182 people (18.7 %) had 1 indicator, and 24 people (2.5 %) had 2 indicators, therefore we dichotomised this variable in to ‘not housing deprived’ and ‘living with (1 or more indicators of) housing deprivation’. The distribution of items that make up the housing deprivation variable are described in Supplementary Tables 6 and 7

**Table 3:** Characteristics of participants included in the mediation analysis, overall and by level of area-based deprivation.

	1 (most deprived)	2	3	4	5 (least deprived)
N	182	200	204	202	188
**age at death, median (IQR)**^b^	74 (64, 81)	76 (64.5, 84)	75 (65, 81)	75.5 (67, 83)	76.5 (66, 82)
**gender**					
Male	95 (52.2 %)	110 (55.0 %)	92 (45.1 %)	110 (54.5 %)	100 (53.2 %)
Female	87 (47.8 %)	90 (45.0 %)	112 (54.9 %)	92 (45.5 %)	88 (46.8 %)
**living alone**					
Not living alone	116 (63.7 %)	130 (65.0 %)	146 (71.6 %)	151 (74.8 %)	143 (76.1 %)
Living alone	66 (36.3 %)	70 (35.0 %)	58 (28.4 %)	51 (25.2 %)	45 (23.9 %)
**housing deprivation**					
Not housing deprived	124 (68.1 %)	143 (71.5 %)	164 (80.4 %)	168 (83.2 %)	171 (91.0 %)
Living with housing deprivation	58 (31.9 %)	57 (28.5 %)	40 (19.6 %)	34 (16.8 %)	17 (9.0 %)
**general health**					
Very good	a (∗∗%)	a (∗∗%)	a (∗∗%)	11 (5.4 %)	10 (5.3 %)
Good	1^a^ (∗∗%)	2^a^ (∗∗%)	2^a^ (∗∗%)	34 (16.8 %)	35 (18.6 %)
Fair	63 (34.6 %)	71 (35.5 %)	64 (31.4 %)	59 (29.2 %)	58 (30.9 %)
Bad	58 (31.9 %)	48 (24.0 %)	57 (27.9 %)	59 (29.2 %)	50 (26.6 %)
Very bad	41 (22.5 %)	49 (24.5 %)	52 (25.5 %)	39 (19.3 %)	34 (18.1 %)
Missing	0 (0.0 %)	0 (0.0 %)	0 (0.0 %)	0 (0.0 %)	1 (0.5 %)
**place of death**					
home	70 (38.5 %)	84 (42.0 %)	80 (39.2 %)	89 (44.1 %)	96 (51.1 %)
hospital	112 (61.5 %)	116 (58.0 %)	124 (60.8 %)	113 (55.9 %)	92 (48.9 %)

a Supressed due to small cell counts (where a number appears before the ∗ (e.g. 1∗) this indicates a partial suppression).

b Interquartile Range (IQR).

#### 4.2.1 Mediation model, preliminary analysis

In the preliminary analysis (Supplementary Table 8) we found that after adjusting for age and sex, people living in more income deprived areas were more likely to live alone and to live with housing deprivation, had worse general health and were more likely to die in hospital, than people who lived in less income deprived areas.

#### 4.2.2 Multiple mediator model

Table 4 shows the results for the Reduced Model and the Full Model. In the Full Model, each reduction in level of area-based deprivation, from quintile 1 (most deprived) to quintile 5 (least deprived), was associated with an odds ratio of 0.88, or a 12 % reduction in the odds of dying in hospital (versus home). Interpreting this in predicted proportions, the marginal results from the Full Model show that 64 % (95 % CI 58 %–69 %) of people living in the most deprived areas (quintile 1) died in hospital, reducing to 51 % (95 % CI 45 %–57 %) for people in the least deprived areas. In the Full Model, people who were living alone were more likely to die in hospital, and people with worse health were less likely to die in hospital; housing deprivation was not associated with death in hospital.

**Table 4:** Multiple mediator model (KHB^b^ method), results for the Reduced and Full logit models showing direct and indirect effects (models also adjusted for age and sex) on death in hospital (versus death at home).

	Reduced Model coef [95 % CI] n = 975	Full Model coef [95 % CI] n = 975	% of total effect due to mediator
**Direct effects:**			
area-based income deprivation¥	−0.12 [-0.21, −0.02]^a^	**−0.13 [-0.22,-0.03]**	–
living alone	–	**0.37 [0.07, 0.68]**	–
housing deprivation	–	−0.07 [-0.60, 0.47]	–
worse general health	–	**−0.32 [-0.44, -0.20]**	–

**Indirect effects of income deprivation¥ via:**			
living alone	–	**−0.01 [-0.03, -0.00]**	12.21
housing deprivation	–	0.00 [-0.01, 0.01]	−1.18
worse general health	–	**0.02 [0.00, 0.04]**	−19.1
			

a ‘total effect’ of income deprivation on death in hospital; ¥ 1 is most deprived, 5 is least deprived.

b Karlson, Holm and Breen (KHB) mediation method.

The last column of Table 4 shows how much of the total effect is explained by the respective mediator; this last column sums to −8.06 which is the overall confounding percentage, in this case a negative number indicating a suppressor effect. Put another way, the effect of income deprivation on death in hospital was 8.06 % bigger in the full mediation model than in the reduced model.

A suppressor effect is present when the inclusion of a mediator (or confounder) increases the effect of an exposure, this can be because the exposure and mediator are correlated but the mediator and exposure have opposite effects on the outcome (Conger, 1974). In our model, the suppressor effect is driven by health because people with worse health were less likely to die in hospital, so after accounting for worse health among people living in more income deprived areas, the effect of income deprivation increases by 19.1 %. Living alone works in the opposite direction to health, attenuating 12.2 % of the total effect of wealth.

In the sensitivity analysis that treated the area-based income deprivation as a categorical variable, worse general health had a statistically significant suppressor effect on the relationship between income deprivation and death in hospital for people living in the most deprived areas (quintile 1) and for those in quintile 3 (Supplementary Table 9). Living alone had a direct effect on the outcome but was not a statistically significant mediator for decedents in any of the deprivation quintiles.

## 5 Discussion

This retrospective observational study of a nationally representative sample of people who died in England and Wales reports novel findings about the relationship between socio-economic position and death in hospital. First, this association is moderated by underlying cause of death. Living in a more income deprived area was associated with death in hospital for people who died from cancer, dementia, and ‘other’ causes, but this association was not observed for people who died from respiratory, cardiovascular, and sudden causes of death. Second, among people who died from cancer, those who lived in more deprived areas were more likely to live alone, and this partly explained why they were more likely to die in hospital. Third, people with worse health were less likely to die in hospital. After accounting for worse health among people living in more deprived areas, living in a more deprived area was more strongly associated with death in hospital.

To our knowledge, this is the first study to test potential moderators of the relationship between area-based deprivation and death in hospital. Like other studies (Davies et al., 2019), we found that people living in more deprived areas were more likely to die in hospital, but our analysis shows that this was not consistent for all causes of death. For respiratory disease, cardiovascular disease, and sudden causes, where the overall rates of death in hospital were higher, we did not see an association between deprivation and death in hospital. These are also the causes of death associated with less access to palliative and end-of-life care (Gadoud et al., 2014; Kendzerska et al., 2019; Sleeman et al., 2015). For cancer deaths, where palliative and end-of-life services are most well established, the social gradient in place of death may indicate that people who are better off, benefit more from the types of services that support people to die at home, an example of the inverse care law (Tudor Hart, 1971). This finding should strengthen calls for services and interventions in palliative and end-of-life care to be designed and evaluated not only for overall benefit but for their distributional effectiveness across subgroups and for how well they close the inequality gap (Cookson et al., 2021).

In this sample and in others (NEoLCIN, 2012), compared to people living in less deprived areas, those living in more deprived areas were less likely to die from cancer and more likely to die from respiratory disease, and this relationship was strengthened after adjusting for differences in age at death. This unequal distribution of underlying cause of death is important to acknowledge because it contributes to the social gradient in hospital deaths seen in the overall sample.

We found that worse health suppressed the effect of area-based deprivation on death in hospital, and therefore, after accounting for worse health among people living in more deprived areas, the association between deprivation and death in hospital was strengthened. This contrasts with findings from our previous study where worse health among people with lower wealth partly explained why they had more hospital admissions in the last year of life (Davies et al., 2021). It may be that for people dying from cancer, worse health could protect against death in hospital, by making prognosis more predictable and planning for death outside of hospital more achievable (Wagner et al., 2010). Methodologically, an awareness of how health can operate differently as a mediator depending on the outcome and population being studied is important for decisions on how to model the relationship between socioeconomic position and health.

In our sample, people living in more deprived areas were more likely to live alone and this explained a small but significant proportion (12.2 %) of the overall effect that living in a more deprived area had on death in hospital. Similar to the findings of a study using the Scottish Longitudinal Study Census data (Schneider, 2019), in our sample, living alone was also more common for women and for older people. Lower life expectancy for men and for people living in more deprived areas may partly explain social patterning in rates of living alone. Recognising living alone as a partial mediator of the relationship between deprivation and death in hospital, highlights a cumulative disadvantage and indicates a potential way for policy makers and practitioners to reduce inequalities in place of death by addressing support for people living alone. For example, through community-based interventions that could provide proportionally more social support to people living alone in more deprived areas. However, more work is needed to understand the type of interventions that would be effective at reducing socioeconomic inequality in place of death.

In our sensitivity analysis, we treated income deprivation as a categorical variable. In this model, living alone was no longer a statistically significant mediator, although the direct effect on death in hospital was still statistically significant. Use of categorical variables can limit statistical power in structural equation models (Wolf et al., 2013), which may explain this loss of statistical significance. Testing this model in a larger sample would be a useful next step for verifying the results.

In our sample of people living at home who died from cancer, those who were living in more income deprived areas were more likely to live with housing deprivation in their last year of life, but housing deprivation did not mediate the relationship between deprivation and death in hospital. This finding contrasts with qualitative work which has highlighted the importance of housing to the care and quality of life of people approaching the end of life (Richards, et al., 2024; Hansford et al., 2022a; Quinn et al., 2023). Our measure of housing deprivation captured a count of items including: overcrowding, lack of central heating, living in shared accommodation, and accommodation type (i.e. not living in a house or bungalow, but living in a flat, tenement, bed-sit, or other). The majority of our sample had no housing deprivation, and very few had more than 1 indicator of housing deprivation, therefore, we dichotomised the sample in to those with no housing deprivation (78.9 %) and those with 1 or more indicators of housing deprivation (21.1 %). Of those with housing deprivation, 76.7 % were classified because of their accommodation type, with a much lower proportion living in overcrowded homes (18.0 %), without central heating (13.1 %), or in shared accommodation (<10 %) (Supplementary Table 6). Therefore, this indicator largely reflects a dichotomy between people living in houses and bungalows and those in flats, tenements and other types of accommodation. Other aspects of housing such as affordability of housing and heating, stability and security of rental contracts, and quality issues including damp, and suitability of kitchen or bathroom facilities are important factors that we were not able to measure in this study (Hansford et al., 2022a; Richards, et al., 2024; Quinn et al., 2023).

## 6 Strengths and limitations

The ONS Longitudinal Study contains high quality and highly complete, self-reported demographic and social data on a nationally representative and sizable sample. A strength of this data is the inclusion of self-reported ethnicity data collected during the Census, considered to be of better quality than ethnicity information contained in medical or administrative records which is often assigned rather than self-identified (Pineda-Moncusí et al., 2024). A limitation of census data is that some groups may be excluded, notably people who are homeless or precariously housed. The level of under-counting is expected to be minimal but is more likely among more socioeconomically deprived groups. Another limitation is the timeliness of the data. The census is carried out every 10 years and there is a delay incorporating the data into the ONS Longitudinal Study. At the time of the analysis, the 2021 Census was not available. Updating the analysis using the 2021 census would be useful for understanding how inequalities may have changed over time.

The ONS Longitudinal Study contains a 1 % sample of people responding to the census providing a relatively large and representative sample. Despite this we did encounter limitations in our analysis due to small sample size. For example, in the moderation analysis, to maintain adequate numbers in cross-sectional categories of deprivation and ethnicity we aggregated some categories of ethnicity which may have masked important differences between these groups. We investigated variation by geographical region; however, this will mask variation within these regions that cannot be explored using the ONS Longitudinal Study. In our mediation model, we focused on a sub-sample of people who died from cancer and were unable to test the mediation model for people who died from dementia, or from ‘other’ causes due to small sample sizes in these subgroups.

We have used robust methods, transparent reporting and tested our results with sensitivity analysis. The findings strengthen our understanding of socioeconomic inequality in place of death and highlight a previously unknown complexity that the social gradient is not seen for all causes of death. A strength of our mediation analysis is that we tested multiple competing pathways in a single model, which may help to reduce bias due to omitted variables (Preacher & Hayes, 2008), however, given the observational nature of our study, we cannot rule out unmeasured confounders which may have biased our results. For example, symptom burden, preferences for care, reasons for terminal hospital admission, access to community-based palliative and end-of-life care, proximity to hospital, and the availability of informal carers are important factors that may have confounded or mediated the direct effect of area-based income deprivation on death in hospital but were not measured in this study. Some of these potential confounders such as proximity to hospital could be investigated with routinely collected mortality data. For others, mortality follow-back studies could be designed to collect the data needed to test these associations.

We used area-based deprivation, based on the postcode of the deceased at the 2011 Census, as a proxy for socioeconomic position. This approach is limited by the ecological fallacy, i.e. it assumes that all people in a neighbourhood will share the same socioeconomic profile. For some people who move house, perhaps to a care home or to a relative's home before death, postcode may be a less good indicator of socioeconomic position, potentially masking some of the social gradient in place of death. Our treatment of place of death assumes that death in hospital is a poor outcome. This is not always the case; hospital may be the preferred or most appropriate place of care and death and many patients die well-supported and with dignity in hospitals (Hoare et al., 2015). However, most people prefer to avoid hospital admissions at the end of their lives, the experience of end-of-life care in hospital is rated lower than in other settings and is associated with worse bereavement outcomes for families (Gomes et al., 2013; Grande & Ewing, 2009; Wright et al., 2010). Therefore, despite offering only a snap-shot of information about the location (not the quality) of care, place of death remains an important quality indicator (Earle, 2003), particularly for studying inequalities because it is captured for the whole population.

## 7 Conclusion

This study contributes to a deeper understanding of *for whom* and *why* socioeconomic inequality in place of death exists. We show for the first time that while overall, people living in more income deprived areas were more likely to die in hospital, this was not consistent for all causes of death. The social gradient in place of death was observed for deaths from cancer, dementia, and ‘other’ causes, but not for deaths from respiratory, cardiovascular, and sudden causes. Our mediation analysis found that for people who died from cancer, after accounting for worse health, the effect of deprivation on death in hospital was strengthened, suggesting that worse health may protect against dying in hospital for some groups. For people who died from cancer, we also found that those living in more deprived areas were more likely to live alone and this partly explained why they were more likely to die in hospital. This novel finding indicates a potential way to reduce inequalities in place of death by addressing support for people living alone. A large proportion of the direct effect of deprivation on death in hospital remains unexplained by the mediators we tested, therefore efforts to understand the causes of this relationship and how to address it should continue. Future studies using linked Census data on larger samples could be used to test mediator models for other causes of death, and to further disaggregate moderators including more detailed categories of ethnicity and different cancer types. An important avenue for future research is to understand how inequality might vary within geographical regions and over time. Datasets should also be developed that capture other potential mediators including access to, and preferences for, healthcare towards the end of life.

## CRediT authorship contribution statement

**J.M. Davies:** Writing – review & editing, Writing – original draft, Visualization, Project administration, Methodology, Investigation, Funding acquisition, Formal analysis, Data curation, Conceptualization. **K.C. Chua:** Writing – review & editing, Supervision, Methodology, Investigation, Conceptualization. **M. Maddocks:** Writing – review & editing, Supervision, Resources, Funding acquisition, Formal analysis. **F.E.M. Murtagh:** Writing – review & editing, Supervision, Methodology, Funding acquisition, Conceptualization. **K.E. Sleeman:** Writing – review & editing, Visualization, Supervision, Methodology, Investigation, Funding acquisition, Formal analysis, Conceptualization.

## Ethical statement

This study was given ethical approval by the curators of the data, the UK Office for National Statistics; study id: 2000705.

## Funding

This study is funded by Marie Curie (MC-21-816). KES is the Laing Galazka Chair in palliative care at King's College London, funded by an endowment from Cicely Saunders International and the Kirby Laing Foundation. FEMM is a UK National Institute for Health and Care Research (NIHR) Senior Investigator. The work is supported by the NIHR Applied Research Collaboration (ARC) for South London. The views expressed in this paper are those of the authors and not necessarily those of the NIHR or the Department of Health and Social Care.

## Competing interests

The authors declare that no competing interests exist; all authors had no financial relationships with any organisations that might have an interest in the submitted work; all authors had no other relationships or activities that could appear to have influenced the submitted work.

## Data Availability

The authors do not have permission to share data.
