# Supplementary material for: Social determinants of where people die: A study of moderators and mediators using linked UK Census and mortality data
Source: SSM Popul Health. 2025 Mar 21;30:101784. doi: 10.1016/j.ssmph.2025.101784 (PMC12005918; doi:10.1016/j.ssmph.2025.101784)
Supplement: Multimedia component 1 [file mmc1.docx]

**Supplementary file for *Davies et al* ‘Social determinants of where people die: A study of moderators and mediators using linked UK Census and mortality data’**

**Table 1: Summary of methods and location of results**

| Stage of analysis | Details of the analysis | Location of results |
| --- | --- | --- |
| Descriptive and preliminary analysis | Description of all variables for overall sample and by quintiles of area-based income deprivation  To understand the relationship between the outcome (place of death) and our main exposure (area-based income deprivation), we used a multinomial logit regression to predict 4 un-ordered categories of place of death (home, hospice, care home; with hospital as the reference category), adjusted for age, sex, and underlying cause of death.  To understand the association between area-based deprivation and cause of death, we report the predicted proportions for each underlying cause of death, by level of area-based deprivation from a multinomial logit regression, adjusted by age and sex. | Manuscript, table 2  Supplementary table 2  Supplementary table 3 |
| Part 1: testing potential moderators | For each potential moderator (age, gender, ethnicity, underlying cause of death, region) we ran 2 models predicting death in hospital (versus home, hospice or care home):   1. A model adjusted for age, sex, deprivation, and the moderator, and 2. A model adjusted for age, sex, deprivation and the moderator, including an interaction term between deprivation and the moderator.   We report the interaction effects.  We also test whether the inclusion of the interaction effects improved the model fit overall using the Wald test (*F* statistic) comparing model i) nested within model ii).  We used Poisson models because risk ratios are arguably more easily interpreted than odds ratios, and applied robust standard errors.  To help interpret the interactions, we graphically depict the marginal effects (i.e. the predicted proportion of death in hospital by level of deprivation for each subgroup of the potential moderator variables) | Supplementary tables 4.1-4.5  Supplementary table 5.  Manuscript, figure 3. |
| Part 2: Multiple mediator model | Description of all variables by quintiles of area-based income deprivation  Preliminary analysis regressing the outcome (death in hospital versus death at home) and each mediator (lives alone, housing deprivation, and general health) on area-based income deprivation using logistic regression or linear regression.  For the multiple mediation model, we used the Karlson, Holm and Breen (KHB) mediation method.  We report results from the *Reduced Model* (i.e. the model reporting the total effect of the exposure) and the *Full Model* (i.e. the model reporting the direct effects of the exposure and the mediators, and the indirect effects of the exposure via the mediators). Our analysis used logit models (the KHB method has not yet been validated for Poisson models) and we have reported coefficients, 95% confidence intervals and the proportion of the confounding explained by each of the mediators. | Manuscript, table 3  Supplementary table 8  Manuscript, table 4 |
| Sensitivity analysis | In a sensitivity analysis we treated the main exposure variable (area-based deprivation) as a categorical variable and discuss differences in the results. | Supplementary table 9 |

**Table 2: Results from multinomial logistic regression using hospital as the base outcome, adjusted for age, sex and underlying cause of death**

| n=34225 | coef | lower 95% CI | upper 95% CI |
| --- | --- | --- | --- |
|  |  |  |  |
| **home** |  |  |  |
| income |  |  |  |
| 1 (most deprived) | **-0.16** | **-0.24** | **-0.07** |
| 2 | **-0.21** | **-0.30** | **-0.12** |
| 3 | -0.06 | -0.15 | 0.02 |
| 4 | -0.04 | -0.13 | 0.05 |
| **hospice** |  |  |  |
| income |  |  |  |
| 1 (most deprived) | **-0.38** | **-0.54** | **-0.22** |
| 2 | **-0.40** | **-0.56** | **-0.24** |
| 3 | **-0.17** | **-0.33** | **-0.01** |
| 4 | -0.01 | -0.17 | 0.14 |
| **care_home** |  |  |  |
| income |  |  |  |
| 1 (most deprived) | **-0.16** | **-0.26** | **-0.06** |
| 2 | **-0.14** | **-0.24** | **-0.04** |
| 3 | 0.06 | -0.03 | 0.16 |
| 4 | 0.04 | -0.05 | 0.14 |

**(data source: ONS LS)**

**Table 3: Predicted proportion of underlying causes of death by level of area-based income deprivation, from multinomial logit model, adjusting for age and sex**

|  | Q1 (most deprived) | Q2 | Q3 | Q4 | Q5 |
| --- | --- | --- | --- | --- | --- |
| cancer | 25.7 [24.7,26.8] | 26.7 [25.6,27.7] | 28.9 [27.8,30] | 30.8 [29.7,31.9] | 31.6 [30.4,32.8] |
| dementia | 8.1 [7.4,8.7] | 8.2 [7.5,8.8] | 7.8 [7.2,8.4] | 8.1 [7.5,8.7] | 8.0 [7.3,8.6] |
| cardiovascular | 25.9 [24.9,27] | 26.7 [25.6,27.7] | 26.4 [25.4,27.5] | 25.4 [24.3,26.4] | 25.7 [24.6,26.8] |
| respiratory | 8.8 [8.2,9.5] | 6.9 [6.3,7.5] | 6.1 [5.5,6.6] | 5 [4.4,5.5] | 4.2 [3.7,4.7] |
| other | 5.2 [4.7,5.8] | 5.1 [4.6,5.6] | 5.5 [5,6.1] | 5.4 [4.9,6] | 5.7 [5.1,6.3] |
| sudden causes | 26.2 [25.2,27.3] | 26.5 [25.5,27.6] | 25.2 [24.2,26.3] | 25.4 [24.3,26.4] | 24.8 [23.7,25.9] |

**(data source: ONS LS)**

**Moderation analysis**

***Notes for tables 4.1-4.5:*** *all models were adjusted for age and sex. Interaction effects are interpreted as the effect (IRR: Incidence Rate Ratio) for each quintile compared to q5 (least deprived), in each category of the moderator compared to the reference group (listed in brakets). For example, the interaction for deprivation and gender, tests whether the effect of being in quintile 1 (compared to quintile 5) is statistically significantly different for women compared to men.*

**(data source for tables 4.1 to 4.5: ONS LS)**

**Table : 4.1 Moderation analysis - Poisson model with interaction effects between area-based income deprivation and age at death, predicting death in hospital (versus death at home, hospice or care home)**

**Table : 4.2 Moderation analysis - Poisson model with interaction effects between area-based income deprivation and gender, predicting death in hospital (versus death at home, hospice or care home)**

**Table : 4.3 Moderation analysis - Poisson model with interaction effects between area-based income deprivation and ethnicity, predicting death in hospital (versus death at home, hospice or care home)**

**Table : 4.4 Moderation analysis - Poisson model with interaction effects between area-based income deprivation and underlying cause of death, predicting death in hospital (versus death at home, hospice or care home)**

**Table : 4.5 Moderation analysis - Poisson model with interaction effects between area-based income deprivation and region of residence, predicting death in hospital (versus death at home, hospice or care home)**

**Table 5: Moderation analysis - model fit statistics comparing Poisson models with and without interactions (see tables 4.1 – 4.5 above), predicting death in hospital (versus death at home, hospice or care home)**

***Notes:*** *models adjusted for age and sex. A statistically significant f statistics (p<0.05) indicates that the inclusion of the interaction term improved the model.*

**(data source: ONS LS)**

| **Model** | **n and *F* statistic** |
| --- | --- |
| Income deprivation and age at death (in years, ref: q5) | n=34225  *F*= 2.26; df(4)  *p*= 0.6881 |
| Income deprivation and sex (ref: q5 and men) | n=34225  *F*=7.59; df(4)  *p*=0.1079 |
| Income deprivation and ethnicity (ref: q5 and white British) | n=34210  *F*=26.94; df(20)  *p*=0.1368 |
| Income deprivation and underlying cause of death (ref: q5 and cancer) | n=34225  *F*=43.81; df(20)  *p*=0.0016 |
| Income deprivation and region (ref: q5 and North East) | n=34225  *F*=28.01; df(36)  *p*=0.8269 |

**Table 6: Breakdown of items that make up the housing deprivation indicator, for those with and without housing deprivation (data source: ONS LS)**

|  | **Not housing deprived** | **Living with housing deprivation** |
| --- | --- | --- |
| N | 770 | 206 |
| **overcrowded** |  |  |
| not overcrowded | 770 (100.0%) | 169 (82.0%) |
| yes, overcrowded | 0 (0.0%) | 37 (18.0%) |
| **central heating** |  |  |
| yes, has central heating | 770 (100.0%) | 179 (86.9%) |
| no, has not got central heating | 0 (0.0%) | 27 (13.1%) |
| **self-contained accommodation** |  |  |
| yes, is self-contained | 770 (100.0%) | 19* (9*%) |
| not self-contained | 0 (0.0%) | ** |
| **housing type** |  |  |
| house or bungalow | 770 (100.0%) | 48 (23.3%) |
| flat, tenement, bed-sit, or other | 0 (0.0%) | 158 (76.7%) |
| **further breakdown of housing type** |  |  |
| detached | 247 (32.1%) | 7 (3.4%) |
| semi-detached | 335 (43.5%) | 25 (12.1%) |
| terraced (including end-terrace) | 188 (24.4%) | 16 (7.8%) |
| block of flats or tenement | 0 (0.0%) | 129 (62.6%) |
| converted or shared house (including bed-sits) | 0 (0.0%) | 1* (*%) |
| commercial building (e.g. office building, hotel, or over a shop) | 0 (0.0%) | ** |
| caravan or other mobile or temporary structure | 0 (0.0%) | ** |

*supressed due to small cell counts (where a number appears before the * (e.g. 1*) this indicates a partial suppression)

**Table 7: Distribution of housing deprivation variable by items of housing deprivation and level of area-based deprivation (data source: ONS LS)**

|  | **1 (most deprived)** | **2** | **3** | **4** | **5 (least deprived)** |
| --- | --- | --- | --- | --- | --- |
| N | 182 | 200 | 204 | 202 | 188 |
| **overcrowded** |  |  |  |  |  |
| no | 169 (92.9%) | 187 (93.5%) | 19* (9*%) | 19* (9*%) | 18* (9*%) |
| yes | 13 (7.1%) | 13 (6.5%) | ** | ** | ** |
| **central heating** |  |  |  |  |  |
| yes, central heating | 17* (9*%) | 19* (9*%) | ** (9*%) | 19* (9*%) | 1** (9*%) |
| no, central heating | ** | ** | ** | ** | ** |
| **self contained accommodation** |  |  |  |  |  |
| yes, self-contained | 18* (9*%) | 19* (9*%) | 204 (100.0%) | 19* (9*%) | 18* (9*%) |
| not self-contained | ** | ** | 0 (0%) | ** | ** |
| **housing type** |  |  |  |  |  |
| house or bungalow | 138 (75.8%) | 155 (77.5%) | 171 (83.8%) | 180 (89.1%) | 174 (92.6%) |
| flat, tenement, bed-sit, or other | 44 (24.2%) | 45 (22.5%) | 33 (16.2%) | 22 (10.9%) | 14 (7.4%) |

**Preliminary analysis regressing the outcome (death in hospital versus death at home) and each mediator (lives alone, housing deprivation, and general health) on area-based income deprivation using logistic regression (shown as OR) or linear regression (shown as coef)**

**Table 8: effect of area-based income deprivation (adjusted for age and sex) on death in hospital (versus death at home) and the three potential mediators**

|  | Lives alone (n=976) | | | Housing deprivation (n=976) | | | General health (n=975) | | | Death in hospital (n=976) | | |
| --- | --- | --- | --- | --- | --- | --- | --- | --- | --- | --- | --- | --- |
|  | OR | lci | uci | OR | lci | uci | coef | lci | uci | OR | lci | uci |
| Age | **1.07** | **1.05** | **1.08** | **1.02** | **1.01** | **1.04** | **-0.01** | **-0.01** | **0.00** | 1.01 | 0.99 | 1.02 |
| women | **2.31** | **1.72** | **3.11** | 1.15 | 0.84 | 1.57 | 0.08 | -0.06 | 0.22 | 1.03 | 0.79 | 1.32 |
| Income deprivation | **0.81** | **0.73** | **0.90** | **0.69** | **0.61** | **0.77** | **-0.07** | **-0.12** | **-0.02** | **0.89** | **0.81** | **0.98** |
|  |  |  |  |  |  |  |  |  |  |  |  |  |
| Marginal effects |  |  |  |  |  |  |  |  |  |  |  |  |
| q1 (most deprived) | 0.36 | 0.30 | 0.41 | 0.34 | 0.28 | 0.39 | 3.65 | 3.53 | 3.76 | 0.63 | 0.57 | 0.68 |
| q2 | 0.31 | 0.27 | 0.35 | 0.26 | 0.23 | 0.30 | 3.57 | 3.49 | 3.66 | 0.60 | 0.56 | 0.64 |
| q3 | 0.26 | 0.23 | 0.30 | 0.20 | 0.17 | 0.22 | 3.50 | 3.43 | 3.57 | 0.57 | 0.54 | 0.60 |
| q4 | 0.23 | 0.19 | 0.26 | 0.14 | 0.12 | 0.17 | 3.43 | 3.34 | 3.52 | 0.54 | 0.51 | 0.58 |
| q5 | 0.19 | 0.15 | 0.23 | 0.10 | 0.07 | 0.13 | 3.36 | 3.24 | 3.49 | 0.52 | 0.46 | 0.57 |

lci: lower confidence interval; uci: upper confidence interval

**(data source: ONS LS)**

**Sensitivity analysis**

**Table 9: KHB model results for the reduced and full logit model (models adjusted for age and sex) and indirect effects on death in hospital (versus death at home)**

|  | reduced model  coef [95% CI]  n=975 | full model  coef [95% CI]  n=975 | % of total  effect due to  mediator |
| --- | --- | --- | --- |
| Income deprivation (q5 is ref) |  |  |  |
| q1 (most deprived) | **0.55 [0.13, 0.97]** | **0.59 [0.16, 1.02]** | - |
| q2 | 0.39 [-0.02, 0.80] | **0.42 [0.01, 0.83]** | - |
| q3 | **0.52 [0.11, 0.93]** | **0.58 [0.17, 0.99]** | - |
| q4 | 0.31 [-0.10, 0.71] | 0.32 [-0.09, 0.73] | - |
|  |  |  |  |
| **Indirect effects** |  |  |  |
| **q1 (most deprived), via** |  |  |  |
| living alone | - | 0.06 [-0.00,0.11] | 9.99 |
| housing deprivation | - | 0.00 [-0.05,0.04] | -0.74 |
| worse health | - | **-0.09 [-0.17,-0.01]** | -16.41 |
| **q2, via** |  |  |  |
| living alone | - | 0.04 [-0.00,0.09] | 11.16 |
| housing deprivation | - | 0.00 [-0.04,0.03] | -0.93 |
| worse health | - | -0.07 [-0.14,0.01] | -17.51 |
| **q3, via** |  |  |  |
| living alone | - | 0.02 [-0.02,0.05] | 3.37 |
| housing deprivation | - | 0.00 [-0.01,0.01] | -0.16 |
| worse health | - | **-0.08 [-0.15,-0.00]** | -15.05 |
| **q4, via** |  |  |  |
| living alone | - | 0.01 [-0.03,0.04] | 2.08 |
| housing deprivation | - | 0.00 [-0.02,0.01] | -0.45 |
| worse health | - | -0.02 [-0.09,0.05] | -6.64 |

**(data source: ONS LS)**
